# Cost-effectiveness of an outreach program for HCC screening in patients with cirrhosis: a microsimulation modeling study

**DOI:** 10.1016/j.eclinm.2025.103113

**Published:** 2025-02-17

**Authors:** Tami Gurley, Ruben Hernaez, Vanessa Cerda, Tynaje Thomas, Manasa Narasimman, Sukul Mittal, Mohammed Al-Hasan, Darine Daher, Amit G. Singal

**Affiliations:** a O’Donnell School of Public Health, University of Texas Southwestern Medical Center, Dallas, TX, USA; b Department of Medicine, Michael E. DeBakey Veterans Affairs Medical Center and Baylor College of Medicine, Houston, TX, USA; c Department of Internal Medicine, University of Texas Southwestern Medical Center, Dallas, TX, USA

**Keywords:** Liver cancer, Screening, Ultrasound, Outreach, Interventions

## Abstract

**Background:**

Patients with cirrhosis are at high risk for hepatocellular carcinoma (HCC), but few undergo guideline-recommended semi-annual screening. Randomized clinical trials (RCTs) demonstrate that mailed outreach can increase screening versus visit-based screening. We estimated the costs and cost-effectiveness of an outreach strategy versus usual care.

**Methods:**

We built a 10-year Markov chain Monte Carlo microsimulation model to conduct a cost-effectiveness analysis comparing a mailed outreach program versus usual care for HCC screening in a cohort of 10,000 patients with cirrhosis. Model inputs were based on literature review (2005—current), and costs were based on inflation-adjusted estimates from Surveillance, Epidemiology, and End Results (SEER)-Medicare claims data. We conducted one-way sensitivity analyses for HCC incidence, outreach costs, efficacy of the outreach strategy to increase screening, and efficacy of curative (versus palliative) HCC treatments.

**Findings:**

Mailed outreach was estimated to cost $32.45 per patient in the first year and $21.90 per patient in subsequent years. The outreach program increased the number of HCC patients detected at an early stage by 48.4% and increased quality-adjusted life years (QALYs) by 300. Cost savings from these increases offset the costs of mailed outreach. Mailed outreach remained cost-effective across a wide range of HCC incidence rates, outreach costs, efficacy of the outreach strategy to increase screening, and the efficacy of curative HCC treatments. Annual out-of-pocket patient costs in the outreach arm were low at $13 per year.

**Interpretation:**

Mailed outreach to encourage HCC screening in patients with cirrhosis dominates usual care and should be considered for implementation in routine practice.

**Funding:**

10.13039/100000054National Cancer Institute and 10.13039/100004917Cancer Prevention Research Institute of Texas.

**Research in context**

**Evidence before this study**

Hepatocellular carcinoma (HCC) screening is associated with improved early tumor detection and overall survival; however, it is underused in clinical practice. Mailed outreach strategies are efficacious and increase HCC screening receipt compared to usual care; however, decision analyses are important to inform health systems about the cost-effectiveness of required institutional investments.

**Added value of this study**

Cost savings from the increased the number of patients with HCC detected at an early stage and increased quality-adjusted life years (QALYs) offset costs of mailed outreach. Mailed outreach remained cost-effective across a wide range of HCC incidence rates, outreach costs, efficacy of the outreach strategy to increase screening, and the efficacy of curative HCC treatments.

**Implications of all the available evidence**

Mailed outreach to encourage HCC screening in patients with cirrhosis is cost-effective across a wide range of parameters. These data support the incorporation of outreach interventions to promote HCC screening into routine clinical practice.

## Introduction

Hepatocellular carcinoma (HCC) is one of the few cancers with a rising mortality rate in the United States and Europe.^1^ Tumor burden at diagnosis is one of the strongest drivers of treatment eligibility and prognosis. Although overall 5-year survival remains below 20%, curative treatment options for patients detected at an early stage afford 5-year survival exceeding 70%.^2^ Guidelines recommend semi-annual HCC screening using abdominal ultrasound with or without alpha-fetoprotein (AFP) in high-risk individuals, including those with non-cirrhotic hepatitis B infection or cirrhosis from any etiology.^3^ HCC screening is supported by a large randomized clinical trial (RCT) among patients with hepatitis B infection and several cohort studies in patients with cirrhosis, showing improved clinical outcomes, including early tumor detection and improved survival.^4^^,^^5^ HCC screening appears to have a favorable risk-benefit ratio with relatively infrequent physical, psychological, and financial harms, and most harms being mild in severity.^6^, ^7^, ^8^

However, there is a gap between HCC screening efficacy in well-controlled settings and effectiveness in clinical practice related to differences in test performance and utilization.^9^ Abdominal ultrasound has impaired visualization and lower sensitivity in obese patients and those with non-viral liver disease—both of which are increasingly common populations undergoing screening.^10^^,^^11^ Further, HCC screening is underused in practice, with only 1 in 4 at-risk individuals undergoing screening, due to a combination of patient- and provider-reported barriers.^12^, ^13^, ^14^, ^15^, ^16^

Several intervention strategies, including provider education, nurse-based protocols, and best practice alerts embedded in electronic health records, have been shown to improve HCC screening utilization.^17^, ^18^, ^19^ However, these strategies only benefit patients actively engaged in routine outpatient care, and over 50% of patients with HCC receive little to no outpatient medical care in the year before their diagnosis.^20^ Therefore, there has been increased interest in population health strategies, such as mailed outreach interventions, that can engage a broader range of patients within a health system.^21^^,^^22^ Mailed outreach strategies are efficacious and cost-effective across several domains, including colorectal cancer screening.^23^, ^24^, ^25^ A multi-site RCT of mailed outreach for HCC screening reported a significant improvement in HCC screening across most examined patient subgroups.^26^ However, mailed outreach programs require institutional investment for both infrastructure and personnel. Herein, we evaluated the cost-effectiveness of implementing a mailed outreach program from both a health system and patient perspective.

## Methods

### Overview of model

Our Markov chain Monte Carlo microsimulation model follows a hypothetical cohort of 10,000 patients with cirrhosis undergoing HCC screening over 10 years. We conducted sensitivity analyses examining results over a 30-year time horizon as well as in larger cohorts of 100,000 and 1,000,000 individuals. The analysis was performed according to the updated Panel on Cost-Effectiveness in Health and Medicine guidelines.^27^ The structure of our model is shown in Supplemental Fig. S1. In brief, we modeled two strategies to implement HCC screening: a mailed outreach strategy and care without an outreach strategy (referred to henceforth as “usual care”). The outreach arm consisted of a one-page letter recommending abdominal ultrasound screenings and reminder phone calls from trained research coordinators, sent to those who did not schedule the screening ultrasound. Usual care consisted of opportunistic recommendations for HCC screening when seen by the patient’s clinical provider.

### Modeling parameters

Key parameters of the model and distributions for probabilistic analyses are described in Table 1. We used inputs for screening completion and HCC detection from two large pragmatic trials of mailed outreach in patients with cirrhosis. The first was an RCT among 1800 patients followed at a single safety-net health system over 18 months (conducted in waves between December 2014 and March 2017), and the second RCT was conducted among 2872 patients who were followed across three health systems over three years (between March 2018 and September 2021).^22^^,^^26^^,^^40^^,^^41^ Details of the study are reported elsewhere,^22^^,^^26^ but in brief, patients with cirrhosis were randomly assigned to receive usual care or mailed invitations for ultrasound-based screening. Patients in both arms experienced the reported natural history of cirrhosis, with a risk of hepatic decompensation and a competing risk of liver-related mortality. In both arms, patients had a chance of incidental detection of HCC at an early stage. Otherwise, adherence to screening informed the likelihood of early HCC detection and curative treatment receipt. False-negative results followed the natural history of HCC until detected incidentally, symptomatically, or by repeat screening examination. Patients with abnormal screening results underwent diagnostic evaluation with contrast-enhanced CT or MRI, with or without subsequent biopsy for confirmation. Those with false-positive screening results returned to ultrasound-based screening after diagnostic resolution.

**Table 1:** Modeling parameters.

	Base Case	Dist.	Range/Std. Dev.	Reference
Proportion of patients with compensated cirrhosis	0.58	Beta	0.05	Tabulation of trial data^26^
Probability of Transition to Decompensated Annual	0.06	Beta	0.02	^28^
Probability of HCC (annual)				
Compensated Cirrhosis	0.02	Beta	0.02	^2^^,^^29^^,^^30^
Decompensated Cirrhosis	0.04	Beta	0.02	^2^
Probability of early detection				
With screening	0.67	Triangle	(0.62, 0.72)	^5^
Without screening	0.33	Triangle	(0.28, 0.38)	^5^
Probability of screening by outreach status				
Baseline	0.23	Triangle	(0.20, 0.27)	^5^^,^^21^^,^^22^^,^^26^
Outreach	0.35	Triangle	(0.32, 0.37)	^5^^,^^21^^,^^22^^,^^26^
Quality of life adjustments				
Compensated cirrhosis	0.8	Triangle	(0.70, 0.9)	31, 32, 33
Decompensated cirrhosis	0.65	Triangle	(0.60, 0.72)	31, 32, 33
Early-stage HCC	0.67	Triangle	(0.55, 0.72)	31, 32, 33
Intermediate/advanced HCC	0.52	Triangle	(0.47, 0.57)	31, 32, 33
Survival (years)				
Compensated cirrhosis	9.8	Triangle	(7.2, 13.0)	34, 35, 36, 37, 38
Decompensated cirrhosis	2.5	Triangle	(0.5, 5.0)	
Early-stage HCC	6.5	Triangle	(1, 13)	
Intermediate/advanced HCC	2	Triangle	(0, 5.5)	
HCC treatment costs (2021 dollars)				
Early-stage HCC	174,981	Gamma	418	^39^
Intermediate/advanced HCC	203,708	Gamma	451	^39^
Outreach costs (2021 dollars)				
Year 1	32.45	Gamma	5.7	Microcosting estimates
Subsequent years	21.9	Gamma	4.7	Microcosting estimates
Discount rate				
Costs	0.03			
QALY	0.03			

For both arms, we accounted for the underlying risk of mortality in patients with cirrhosis and the increased risk of HCC in patients with decompensated cirrhosis. Patients diagnosed with HCC were assigned utilities, anticipated survival, and costs related to tumor stage at diagnosis. For patients with undetected HCC, either due to lack of surveillance or a false negative result, we modeled a risk of HCC progression and decreased likelihood of detecting the HCC at an early stage. For those without HCC, the utilities and costs were driven by the presence of compensated versus decompensated cirrhosis.

### Cost accounting of the outreach intervention

Outreach costs are calculated using micro-costing to track time spent on chart review for program eligibility and patient outreach activities. Project staff tracked activities and time spent during the trial. Additionally, we include one-time costs for implementation of the outreach infrastructure (e.g., computer set-up, language validation) and ongoing operation costs. Costs specifically related to research activities are excluded from the cost estimates, as those would not be encountered if outreach were adopted in clinical practice. Average implementation and maintenance costs were calculated on an annual per-patient basis. We also estimated ongoing program costs for enrolled patients, which excludes chart review for eligibility and program development expenses.

### Patient costs

We additionally considered the costs of screening from a patient perspective, including costs of patient travel and time off from work for ultrasound-related medical appointments. We assumed no additional indirect costs for AFP testing, as this is typically done at the same time as the patient’s clinic visit. Patients who received ultrasound screening incurred travel costs ($0.16 per mile for 20 miles roundtrip) and lost wage costs based on a 30% employment rate and 3 h. Lost wages were valued at the mean 2021 Dallas area wage rate and a 27.6% benefits rate.^42^, ^43^, ^44^

### Cost-effectiveness

We calculated the difference in quality-adjusted life years (QALY) between the mailed outreach strategy and usual care. We calculated the incremental cost-effectiveness ratio (ICER) for the two strategies and interpreted the results concerning the contemporary willingness of pay threshold of $100,000 per QALY. We conducted a series of one-way sensitivity analyses for HCC incidence, outreach costs, differences in screening adherence (i.e. efficacy of outreach), and efficacy of curative HCC treatment (versus palliative therapies). All estimates are presented in 2021 dollars using the Consumer Price Index Retroactive Series and include a 3% discount rate. Cost-effectiveness analyses were completed in Amua software.^45^

### Ethics

The RCTs which served as inputs for this cost-effectiveness analysis were approved by the IRBs of UT Southwestern Medical Center and the Michael E. DeBakey VA. For both RCTs, a waiver of informed consent was obtained to avoid volunteer bias.

### Role of funding source

Funding sources had no role in study design, data collection, data analysis, interpretation, or writing of the report. The content is solely the responsibility of the authors and does not necessarily represent the official views of the NIH, CPRIT, or the US government. All authors had access to the data and accept the responsibility to submit for publication after approving the final draft.

## Results

### Base case outcomes

**Fig. 1:**
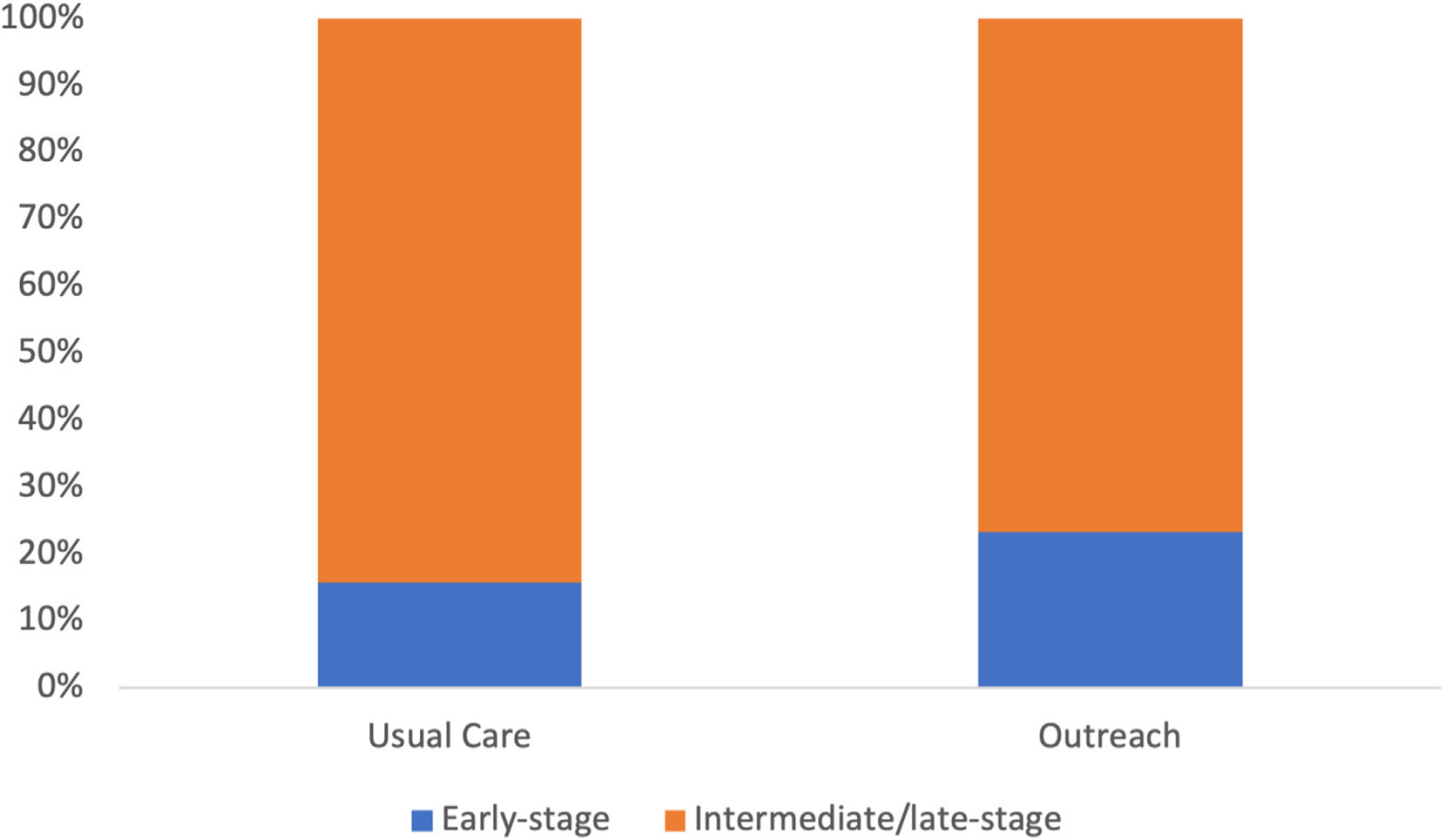
Proportions of patients with early-stage HCC in mailed outreach and usual care arms. Early-stage HCC detection over 10 years is higher in the outreach arm compared to usual care.

We modeled a 2% annual HCC incidence in patients with compensated cirrhosis and a 4% annual incidence in those with decompensated cirrhosis, resulting in 602 anticipated HCC among 10,000 patients over 10 years. Based on a literature review of screening receipt for usual care and results from prior RCTs evaluating mailed outreach,^5^^,^^21^^,^^22^^,^^26^ the probability of screening receipt was modeled at 23% in the usual care arm and 35% in the outreach arm (Table 1). Mailed outreach resulted in a 48.4% relative increase in early-stage HCC diagnosis, with 223 patients in the mailed outreach arm and 331 in the usual care arm detected at an early stage (Fig. 1).

### Outreach costs

The empirical costs of implementing a mailed outreach are presented in Table 2. Overall, the cost of a mailed outreach intervention was $32.45 per patient in the first year and approximately $21.90 per patient for each additional year. The higher costs in the first year were largely driven by the need for chart review to confirm the presence of cirrhosis and excluded patients in whom screening was not recommended (e.g., Child-Turcotte-Pugh C cirrhosis); this process was only needed for patients new to the health systems and therefore less time intensive in subsequent years. Outreach costs were otherwise primarily driven by staff time dedicated to outreach activities (phone calls, mailings), with program coordination and computer support being the next biggest cost categories. Annual costs for postage and outreach materials were less than $2 per patient.

**Table 2:** Costs of mailed outreach.

Outreach Component	Hours	Total Cost	Costs Per Patient
Nurse Practitioner (screen patients for eligibility)^a^	289	$22,318.29	$15.54
Program Management (coordinate supplies, train staff)^b^	50	$2377.29	$1.66
Computer/System support	40	$1170.05	$0.81
Language transcription/translation	10	$282.13	$0.20
Language validation	5	$141.06	$0.10
Prep recruit materials	20	$545.22	$0.38
Supplies for letters			
Postage		$2369.40	$1.65
Materials (envelopes, labels, paper, printing)		$340.12	$0.24
Patient outreach (staff time for calls and mailing)	1040	$40,361.76	$28.11
Total costs for HCC outreach		$69,905.32	$48.68
Total patients in trial			2872
Number of patients in outreach arm			1436
Cost per patient for outreach			$48.68
Annual cost per patient–first year			$32.45
Annual cost per patient -additional years			$21.90

All amounts are in 2021 dollars and reflect 18 months of outreach per patient unless otherwise noted.

a We assumed 10 min per chart to identify eligible patients and exclude those in whom screening is not recommended (e.g., Child C Pugh cirrhosis or significant co-morbid conditions). We assumed an hourly wage of $64.25 and fringe rate of 30%, which was adjusted for 2021 dollars using the Consumer Price Index Research series.

b Salaries for program management, research assistance, computer specialist and language services are 2021 national average salaries reported by the US Bureau of Labor Statistics.

### Cost-effectiveness

Mailed outreach strongly dominated usual care as it resulted in a net cost savings of $1.9 million per 10,000 patients from a health system perspective and it resulted in 300 more QALY over ten years (Table 3). Results were similar for a time horizons from 5 to 30 years (cost savings of $151 to $202 per patient and 251 to 326 additional QALYs per 10,000 patients) as well as cohort sizes from 5000 to 1,000,000 (cost savings of $177 to $189 per patient and 284 to 304 additional QALYs per 10,000 patients).

**Table 3:** Cost-effectiveness of mailed outreach.

	Arm	Cost	QALY	Per-patient cost	Per-patient QALY
Base case					
10,000 patients over 10 years	Outreach	$251,061,342	31,901	$25,106	3.19
	No outreach	$252,912,367	31,601	$25,291	3.16
	Difference	−$1,851,025	300	−$185	0.03
Sensitivity analyses					
10,000 patients over 5 years	Outreach	$210,124,334	27,204	$21,012	2.72
	No outreach	$211,639,195	26,953	$21,164	2.70
	Difference	−$1,514,861	251	−$151	0.03
10,000 patients over 15 years	Outreach	$264,869,267	33,568	$26,487	3.36
	No outreach	$266,830,131	33,250	$26,683	3.33
	Difference	−$1,960,864	318	−$196	0.03
10,000 patients over 20 years	Outreach	$269,670,083	34,179	$26,967	3.42
	No outreach	$271,667,906	33,856	$27,167	3.39
	Difference	−$1,997,823	323	−$200	0.03
10,000 patients over 30 years	Outreach	$272,094,155	34,497	$27,209	3.45
	No outreach	$274,110,514	34,171	$27,411	3.42
	Difference	−$2,016,359	326	−$202	0.03
5000 patient cohort over 10 years	Outreach	$126,510,950	15,943	$25,302	3.19
	No outreach	$127,395,203	15,797	$25,479	3.16
	Difference	−$884,253	146	−$177	0.03
15,000 patient cohort over 10 years	Outreach	$376,969,324	47,760	$25,131	3.18
	No outreach	$379,780,240	47,305	$25,319	3.15
	Difference	−$2,810,916	455	−$187	0.03
50,000 patient cohort over 10 years	Outreach	$1,260,078,952	160,158	$25,202	3.20
	No outreach	$1,269,457,924	158,638	$25,389	3.17
	Difference	−$9,378,972	1520	−$188	0.03
*100,000* patients over 10 years	Outreach	$2,524,894,694	319,927	$25,249	3.20
	No outreach	$2,543,769,971	316,876	$25,438	3.17
	Difference	−$18,875,277	3051	−$189	0.03
*1,000,000* patients over 10 years	Outreach	$25,307,632,507	3,197,122	$25,308	3.20
	No outreach	$25,496,349,828	3,166,625	$25,496	3.17
	Difference	−$188,717,321	30,497	−$189	0.03

QALY—quality adjusted life year.

**Fig. 2:**
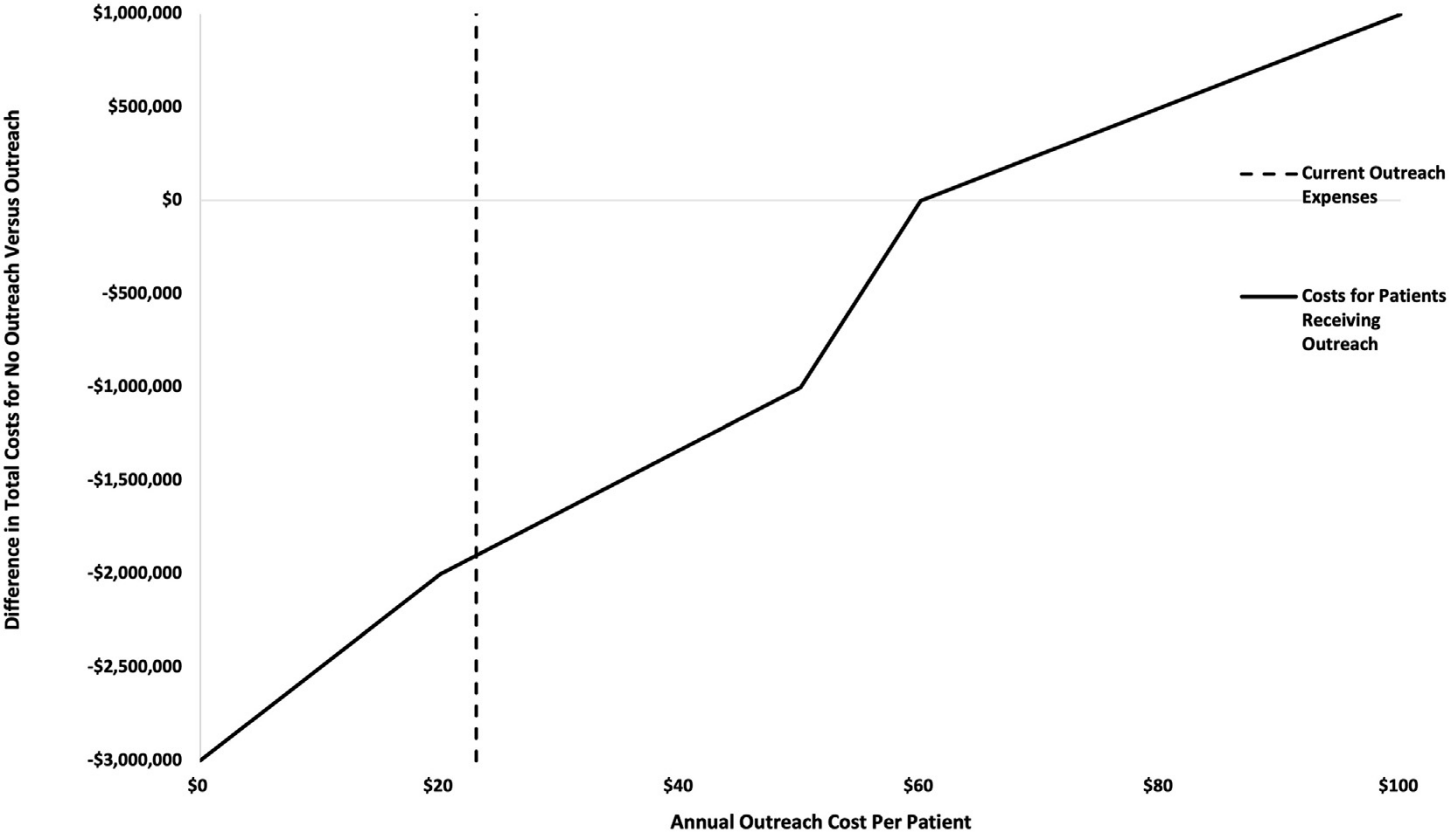
One-way Sensitivity Analysis for Cost of Mailed Outreach. In one-way sensitivity analyses, mailed outreach remained cost-saving until outreach expenses exceeded $60 per patient per year.

**Fig. 3:**
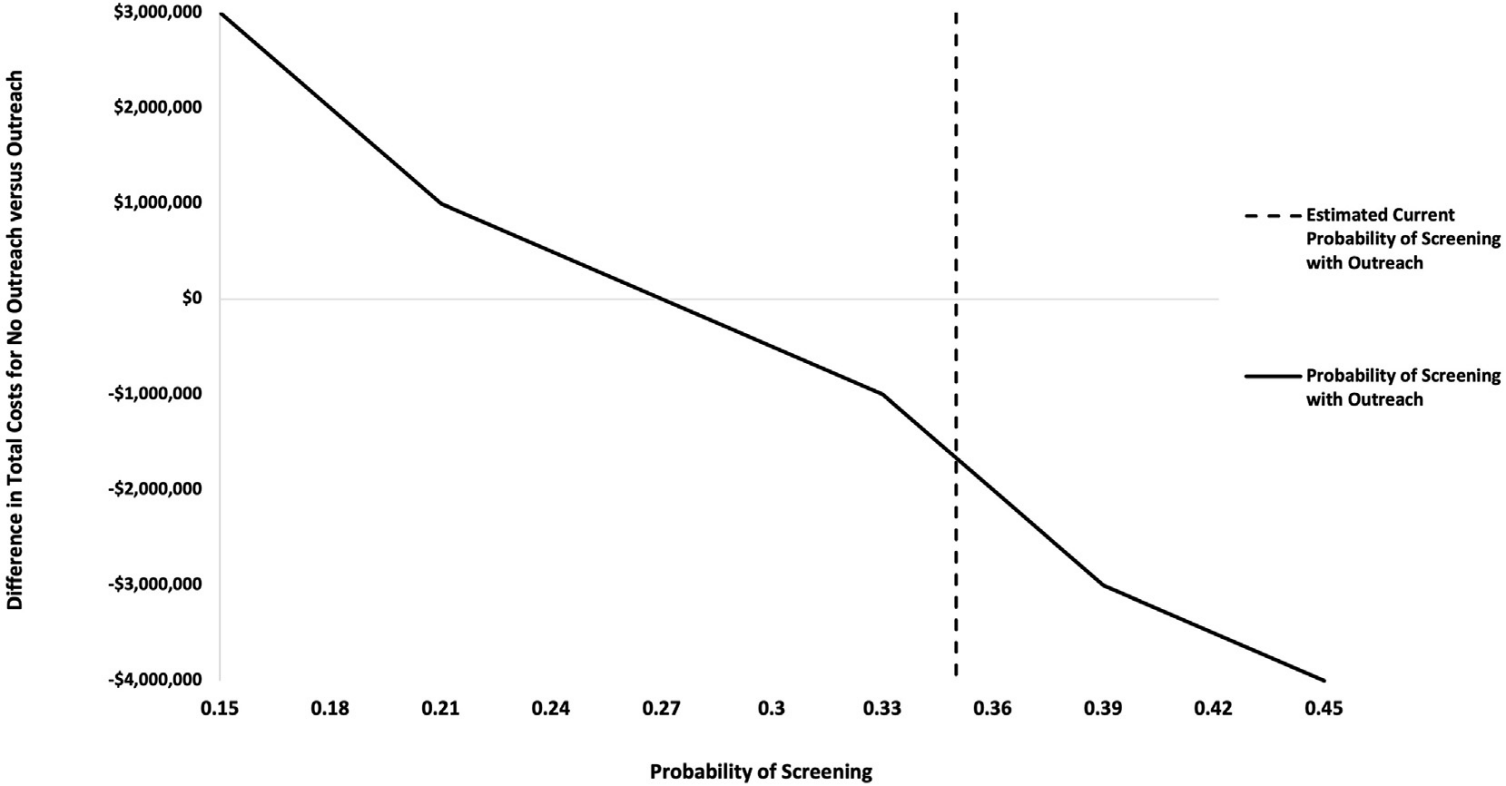
One-way Sensitivity Analysis for Effectiveness of Mailed Outreach. In one-way sensitivity analyses, mailed outreach was cost savings with increases in the probability of screening >5% compared to usual care.

In one-way sensitivity analyses, mailed outreach remained cost-saving until outreach expenses exceeded $60 per patient per year, i.e., nearly 2-fold greater than estimated costs in year 1 and nearly 3-fold greater than estimated ongoing annual costs (Fig. 2). When varying potential effectiveness estimates, mailed outreach remained cost-saving after increases in screening adherence >5% compared to usual care (Fig. 3). Any increase in screening resulted in increased QALY, and the ICER for an increase of only 3% compared to usual care (i.e., 22% versus 25%) was $12,373. Mailed outreach was cost-effective across a wide range of annual incidence rates but gains were greater at higher incidence rates. Mailed outreach was cost-saving for annual rates exceeding 1% in patients with compensated cirrhosis and all examined rates (i.e, ≥2%) for those with decompensated cirrhosis. Finally, mailed outreach yielded increased QALYs as long as the average expected survival from early detection was at least 5 months greater than intermediate/late-stage diagnosis. From a patient perspective, annual out-of-pocket patient costs were estimated to be $13 per year.

## Discussion

Several studies have highlighted the widespread underuse of HCC screening in clinical practice, underscoring the importance of intervention strategies to improve early-stage HCC detection.^12^ RCTs demonstrate the efficacy of mailed outreach to increase HCC screening^22^^,^^26^; however, this approach can be costly for health systems and there have been limited data examining cost-effectiveness. Our study demonstrated that the costs of mailed outreach were offset by cost savings related to early-stage HCC detection, resulting in cost savings of over $250,000 and 140 more QALYs among 10,000 patients over 10 years. These data underscore the value of interventions, such as mailed outreach, to promote HCC screening in large health systems.

Notably, mailed outreach remained cost-effective across a wide range of inputs for HCC incidence rates and the efficacy of the outreach strategy. These data are important given the changing epidemiology of at-risk populations, with an increasing proportion of patients with alcohol-related liver disease and metabolic dysfunction associated steatotic liver disease.^29^ Although patients with cirrhosis from any etiology have a sufficient annual risk of HCC to warrant screening, patients with non-viral liver disease have a lower risk than those with viral liver disease.^46^ Similarly, mailed outreach was shown to be efficacious in several studies although the relative benefit can differ across health systems, including greater benefit in safety-net health systems and large integrated systems in which HCC screening through usual care is lower than tertiary care referral centers.^26^

Mailed outreach also remained cost-effective across a wide range of inputs for HCC treatment efficacy; however, it was no longer cost-effective when the clinical benefits of early-stage detection and curative treatment were mitigated.^47^ This situation may apply to patient populations with limited access to curative surgical treatments as well as environments where the efficacy of palliative treatments improves.^48^ The introduction of immune checkpoint inhibitors alone or in combination with locoregional therapy can offer significantly improved survival for those with tumors beyond an early stage, with a portion of those patients being downstaged to surgical therapies including liver transplantation.^49^^,^^50^

Although mailed outreach is efficacious, studies report semi-annual HCC screening completion in less than 25% of patients. These data highlight that outreach alone is likely insufficient to overcome all barriers to screening completion. A prior study among >1000 patients with cirrhosis found that nearly two-thirds failed to have regular outpatient care in the year before HCC diagnosis.^20^ Although the most common barrier among patients who engaged in routine care was related to a lack of provider orders, there are other downstream barriers, including patient non-adherence, mitigating semi-annual screening completion.^51^ According to a recent study, financial incentives were not found to be beneficial, however other concomitant interventions, such as patient education or navigation, could be beneficial.^52^ If those were shown to be efficacious, updated cost-effectiveness analyses could help inform the value to health systems for adoption.

Finally, we found that mailed outreach is associated with minimal out-of-pocket costs for patients. Most data regarding the financial burden of cancer care has focused on those with known cancer, although recent data have also highlighted the potential for the possible financial impact of HCC screening.^8^ Indeed, patients cite screening costs as a barrier to HCC screening completion, which consequently can disproportionately impact imaging-based screening.^15^ A study using the Optum database reported substantial variation in out-of-pocket costs, with higher costs observed in patients who underwent MRI-based screening than ultrasound-based screening.^53^ Conversely, indirect costs can be mitigated and adherence could be increased with emerging biomarker-based screening strategies, which can be done on the same day as a clinic visit.^54^

Our results should be interpreted considering their limitations. First, modeling studies inherently depend on study inputs and assumptions; we used existing contemporary literature including triangulating data from several sources when possible. To account for uncertainty in some inputs, we evaluated our results in one-way sensitivity analyses and results were consistent across a wide range of estimates for implementation costs, HCC incidence, and outreach efficacy to increase HCC screening. Estimates were also robust to a range of cohort sizes and time horizons. Second, our results assume ultrasound-based screening, which may not apply to emerging imaging- and blood-based strategies.^55^, ^56^, ^57^ Third, all studies evaluating mailed outreach were conducted in select health systems and may not apply to all patient populations, particularly those outside the United States.

In conclusion, we demonstrated that mailed outreach is cost-effective across a wide range of parameters. Our data underscore the importance of incorporating outreach interventions into routine clinical practice to promote HCC screening implementation.

## Contributors

The authors confirm contribution to the paper as follows:

Study conception and design: AS, TG.

Data analysis: TG.

Interpretation of results: all authors.

Draft manuscript preparation: TG, AS.

Critical revision of the manuscript: all authors.

All authors reviewed the results and approved the final version of the manuscript.

Drs. Singal and Gurley had full access to all data in the study and take responsibility for the integrity of the data and accuracy of the data analysis.

## Data sharing statement

Data supporting the findings of this study are available within the article and its Supplemental materials. Other study materials and data related to the study are available from the corresponding author, upon reasonable request.

## Declaration of interests

Amit G. Singal has served as a consultant or on advisory boards for Genentech, AztraZeneca, Eisai, Bayer, Exelixis, Elevar, Merck, Boston Scientific, Sirtex, HistoSonics, FujiFilm Medical Sciences, Exact Sciences, Roche, Glycotest, Abbott, Freenome, and GRAIL.

None of the other authors have any relevant conflicts of interest.
