# Supplementary figures and images for: Cost-effectiveness of an outreach program for HCC screening in patients with cirrhosis: a microsimulation modeling study
Source: eClinicalMedicine. 2025 Feb 17;81:103113. doi: 10.1016/j.eclinm.2025.103113 (PMC11876903; doi:10.1016/j.eclinm.2025.103113)

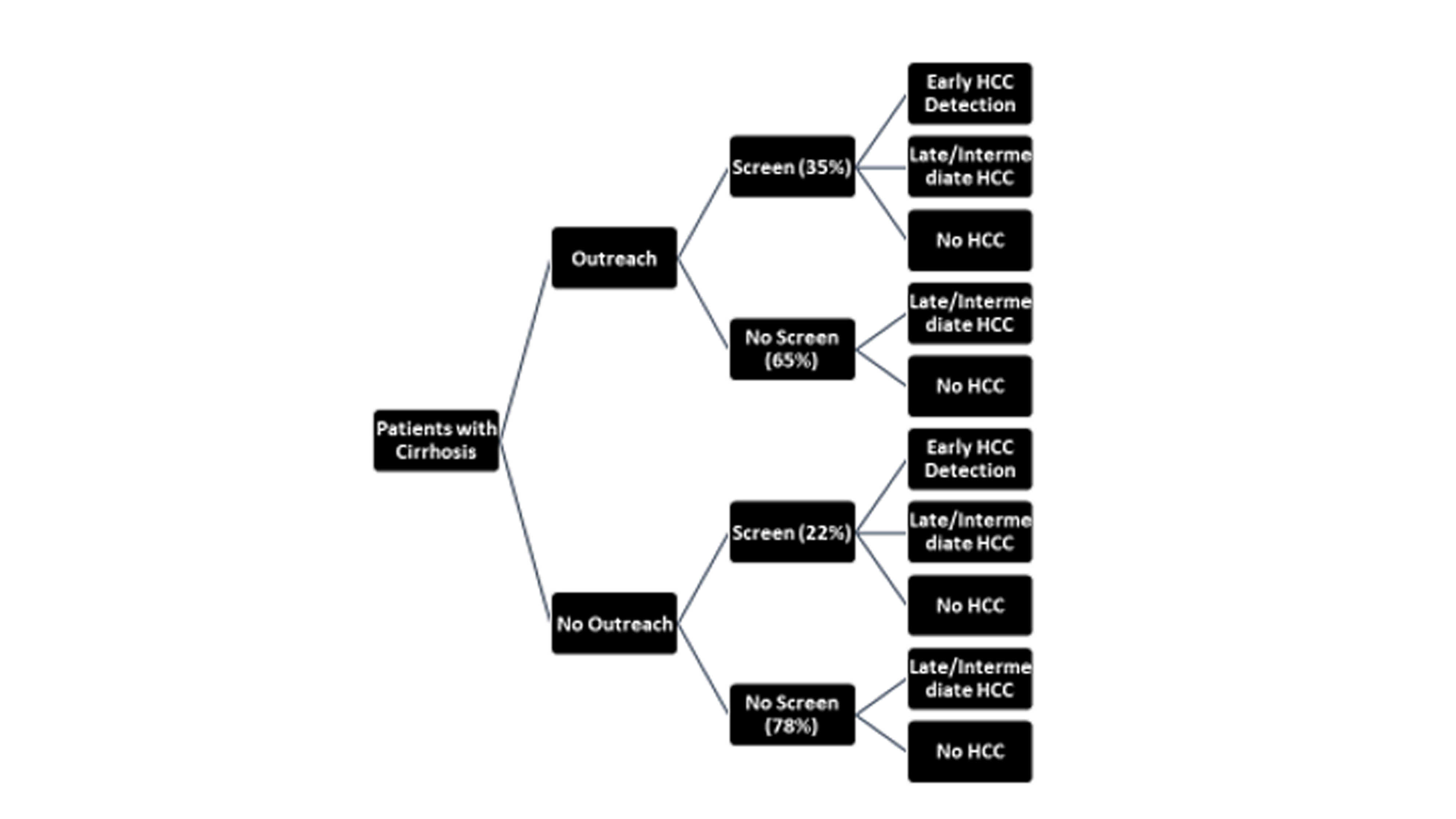

Supplement: Supplemental Fig. S1 [file figs1.jpg]
